# Structural and Functional Characterization of Deceased Donor Stem Cells: A Viable Alternative to Living Donor Stem Cells

**DOI:** 10.1155/2019/5841587

**Published:** 2019-11-25

**Authors:** Prakash N. Rao, Dayanand D. Deo, Misty A. Marchioni, Rouzbeh R. Taghizadeh, Kyle Cetrulo, Sharyn Sawczak, Jacob Myrick

**Affiliations:** ^1^NJ Sharing Network, New Providence, NJ, USA; ^2^AuxoCell Laboratories, Inc., Cambridge, MA, USA

## Abstract

Stem cells can be isolated from various human tissues including bone marrow (BM) and adipose tissue (AT). Our study outlines a process to isolate adult stem cells from deceased donors. We have shown that cell counts obtained from deceased donor BM were within established living donor parameters. Evaluation of demographic information exhibited a higher percentage of hematopoietic stem cells (HSC) in males versus females, as well as a higher percentage of HSC in the age bracket of 25 years and under. For the first time, we show that deceased donor femur BM grew cell colonies. Our introduction of new technology for nonenzymatic AT processing significantly increased cell recovery over the traditional enzymatic processing method. Cell counts from the deceased donor AT exceeded living donor parameters. Furthermore, our data illustrated that AT from female donors yielded a much higher number of total nucleated cells (TNC) than males. Together, our data demonstrates that our approach to isolate stem cells from deceased donors could be a routine practice to provide a viable alternative to living donor stem cells. This will offer increased accessibility for patients awaiting stem cell therapies.

## 1. Introduction

Stem cells are an integral part of regenerative medicinal applications [1]. In order to be a viable therapeutic alternative, stem cells should be available in abundant quantities capable of being harvested by minimally invasive procedures, easily transplanted to either an autologous or allogeneic host, and be differentiated along multiple cell lineage pathways in a regulated and reproducible manner [2].

Adult stem cells, found in a host of tissues throughout the body, are a viable option for clinical use due to their flexibility in their differentiating capacity. They can be categorically divided into hematopoietic stem cells (HSC), mesenchymal stem cells (MSCs), and tissue-specific stem cells. The three most common sources for adult stem cells are the bone marrow, peripheral blood, and adipose tissue [3].

There are many patients awaiting a life-saving stem cell transplant who do not have a suitable donor. Suitability of HSC donors is determined by the matching of a genetically inherited tissue type. Matching tends to occur most within donors and patients who have similar racial/ethnic backgrounds. This can make finding a suitable stem cell donor difficult, if not impossible, for patients whose racial/ethnic background is currently underrepresented in the national donor registry [4].

Bone marrow has been considered the common source of adult stem cells procured from living donors and is primarily used for hematopoietic reconstitution after myeloablative therapy to treat cancers, leukemia, strong anemias, and some genetic disorders [5, 6]. HSC can also be mobilized from the bone marrow and harvested from peripheral blood. The presence of MSC in bone marrow has also been observed at a very low percentage [7].

Adipose tissue is a rich source of MSC which reside in the stromal vascular fraction (SVF) during the isolation process [8–10]. The low-morbidity extraction procedure through liposuction and high yield of MSC make human adipose tissue a readily available source of stem cells [11].

Stem cells for clinical use are currently only procured from living donors, limiting the number of available products. The extraction of stem cells from living donors is subject to limited volumes, cell counts, and discomfort to the donor. HSC transplants, in addition to being compatible, need to have a high enough cell yield in order to be considered sufficient for transplantation. This yield is based on a minimum cell dose per patient weight.

The procurement of stem cells from other sources beside living donors is a true possibility that needs to be explored [12]. Obtaining organs and tissues for transplantation from deceased donors is a widely accepted strategy; however, during the routine deceased donor process, procuring the bone marrow and adipose tissue is not performed. Deceased donor bone marrow and adipose tissue can be procured, substantially increasing the supply and access to stem cells without the pain, morbidity, and mortality associated with living donor stem cell collections [13].

The NJ Sharing Network is a nonprofit, federally designated organ procurement organization responsible for the recovery of organs and tissues for patients awaiting transplantation and is uniquely positioned to obtain both bone marrow and adipose tissue from research-consented deceased donors. In this study, we describe the process of obtaining and characterizing stem cells from deceased donors that can be routinely recovered for regenerative medicine procedures. These cells can be cryopreserved and/or *ex vivo* expanded for current or future therapeutic applications [14–17]. In addition, we have developed a new technique for nonenzymatic isolations of MSC from deceased donor adipose tissue, thus significantly increasing the number of viable cells obtained.

## 2. Materials and Methods

### 2.1. Patient Demographics

We identified 33 research-consented deceased donors from our local service area (19 males; 14 females) prior to their organ procurement workup. Their ages ranged from 13 to 69 years with races broadly distributed among the local population (13 Caucasians, 6 Black, 13 Hispanic, and 1 South Asian). The determination of tissue collection was based on clinical and/or technical reasons during the deceased donor workup. Causes of death include stroke, drug intoxication, motor vehicle accident (MVA), suicide, head trauma, cardiac arrest, homicide, and other natural causes (Table 1).

### 2.2. Bone Marrow

Extraction of iliac crest bone marrow from the deceased donors was performed using a Fenwal™ Bone Marrow Collection Kit (Fenwal Inc., Lake Zurich, IL, USA), an aspiration needle, and a heparinized syringe. The bone marrow was expelled into the collection bag portion of the bone marrow collection kit. The aspiration needle was then moved to a different site on the iliac crest for further collection. Bone fragments were filtered out via the gravity filtration system of the bone marrow collection kit. The collected bone marrow unit was shipped to the laboratory on wet ice.

Extraction of femur bone marrow from the deceased donor was performed using a bone saw, a Fenwal™ Bone Marrow Collection Kit, and a heparinized syringe. The femur was removed and the shaft was cut at both ends to reveal the bone marrow. Heparin was flushed into the shaft, and the bone marrow was expelled into the collection bag portion of the bone marrow collection kit. Bone fragments were filtered out via the gravity filtration system of the bone marrow collection kit. The collected bone marrow unit was shipped to the laboratory on wet ice.

In the laboratory, the collection was divided into aliquots and centrifuged at 800 x g for 10 minutes at room temperature. The buffy coat layer, containing the mononuclear cells including HSC, was carefully extracted. A portion of the well-mixed buffy coat cell suspension was used for immunostaining to detect the expression of cell surface markers CD34 and CD45.

The cell surface markers were detected on a BD FACSCanto™ II (BD Biosciences, San Jose, CA, USA) using a BD™ Stem Cell Enumeration Kit (BD™ SCE kit) containing process controls. The BD™ SCE kit provides a single tube assay for the detection of viable CD34+ cells in fresh bone marrow. Briefly, the reagent was combined with test samples (buffy coat) in individual BD Trucount™ Tubes to obtain absolute cell counts. The sample was added to the reagent according to the manufacturer's instructions, thus allowing the fluorochrome-labeled antibodies in the reagent to bind specifically to the surface of the HSC. The dye 7-aminoactinomycin D (7-AAD) was added to assess the viability of the cells. Erythrocytes were lysed using ammonium chloride before the sample was acquired on the flow cytometer. The concentration of viable CD34+ cells, viable CD45+ cells, and the percentage of viable CD34+ cells in the viable CD45+ cell population in the sample was identified [18].

### 2.3. Colony-Forming Unit (CFU) Assay

The CFU assay for the bone marrow HSC from the iliac crest and femur was carried out by first determining the volume of bone marrow buffy coat required to plate cells at a density of 1.25 × 10^4^ cells per well. Contamination of red blood cells was minimized by sedimentation over HetaSep™ (Stemcell Technologies Inc., Vancouver, BC, Canada). A 1 ml working solution containing 10x cell suspension was prepared using Iscove's Modified Dulbecco's Media (IMDM) (Stemcell Technologies Inc., Vancouver, BC, Canada). To setup the CFU assay, 400 *μ*l of the 10x working cell suspension was added to 4 ml of prealiquoted and thawed MethoCult™ media (Stemcell Technologies Inc., Vancouver, BC, Canada). The MethoCult™/cell mixture was dispensed in 1 ml aliquots into the prelabeled SmartDish™ (Stemcell Technologies Inc., Vancouver, BC, Canada) in triplicate. Sterile water was added to the space between the wells to help maintain humidity during incubation. The SmartDish™ containing the MethoCult™/cell mixture and water was incubated in a CO_2_ incubator set at 37°C and 5% CO_2_ for 14-16 days. After the incubation period, each distinct colony was counted and identified by its specific morphological characteristics using an inverted phase contrast microscope.

### 2.4. Adipose Tissue

Adipose tissue was excised from the abdomen of the deceased donor and placed in a sterile transport container. The container was transported to the laboratory on wet ice.

In the laboratory, two equal mass fractions of adipose tissue were collected, minced, and processed in individual one-time use, disposable AC:Px® Systems (AuxoCell Laboratories, Inc., Cambridge, MA, USA). The finely minced tissue was washed with 0.9% sodium chloride (B. Braun, Bethlehem, PA, USA) saline. The minced tissue product from Fraction 1 was treated with Collagenase Type II (Thermo Fisher Scientific, Waltham, MA, USA) enzyme at a concentration of 150 *μ*g/ml and agitated in a 37°C incubator for 1 hour. The minced tissue from Fraction 2 was processed in a similar manner to Fraction 1 except that no enzyme was added to Fraction 2. After 1 hour of incubation, both fractions were filtered and processed through the series of bags of the AC:Px® System and centrifuged at 430 x g for 30 minutes. The cell pellet for each fraction was resuspended in PBS (Phosphate Buffered Saline; Thermo Fisher Scientific, Waltham, MA, USA) to a final volume of 20 ml and represented the SVF for Fraction 1 (enzyme treated) and Fraction 2 (nonenzyme treated).

The minimal criteria for the phenotyping of MSC are the expression of cell surface markers CD73, CD90, CD29, CD44, and CD105 accompanied by the lack of expression of CD11b, CD34, CD45, CD79a, and HLA-DR [19]. Literature suggests that the expression of CD34 on adipose-derived MSC is controversial and may show up in varying degrees [20–24]. A cell count for the SVF was performed on a Guava easyCyte™ HTS flow cytometer (Luminex, Austin, TX, USA) using the ViaCount™ assay reagent as per manufacturer's instructions. Well-mixed samples were taken and aliquoted in separate tubes for antibody staining. Antibodies used in our study were APC-conjugated mouse anti-human CD73, PerCP-Cy™5.5-conjugated mouse anti-human CD105, PE-conjugated mouse anti-human CD44 (BD Stemflow™ Human MSC Analysis Kit; BD Biosciences, San Jose, CA, USA), APC-conjugated mouse anti-human CD90, PE-conjugated mouse anti-human CD29, FITC-conjugated mouse anti-human CD45, and FITC-conjugated mouse anti-human CD11b/MAC-1 (BD Pharmingen; BD Biosciences, San Jose, CA, USA). All the above antibodies were added to the aliquoted samples and incubated in the dark for 30 minutes at room temperature. The cells were washed twice with wash buffer (PBS containing 1% Fetal Bovine Serum) and resuspended in wash buffer for analysis on the flow cytometer. The samples were gated on cells negative for FITC (CD11b/MAC-1, CD45) and positive for APC (CD73, CD90), PerCP-Cy™5.5 (CD105), and PE (CD44, CD29).

### 2.5. Cell Growth and Proliferation

MSCs from the nonenzyme-treated Fraction 2 SVF were grown in a CELLstart™ CTS™ (Thermo Fisher Scientific, Waltham, MA, USA) coated flask as follows. CELLstart™ CTS™ was diluted 1 : 100 in 10 ml PBS and added to a 75 cm^2^ tissue culture flask (Falcon®, Corning, Corning, NY, USA) gently swirled to ensure complete surface coverage. The flask was incubated in a humidified CO_2_ incubator set at 37°C and 5% CO_2_ for 60 minutes, then placed in a laminar floor hood until use. Before adding the cells, the CELLstart™ CTS™ solution was aspirated and replaced with a StemPro® MSC SFM CTS™ complete growth medium (Thermo Fisher Scientific, Waltham, MA, USA) containing 2% L-glutamine (Sigma-Aldrich, St. Louis, MO, USA) and 1% antibiotic (Penicillin-Streptomycin; Thermo Fisher Scientific, Waltham, MA, USA). The volume of SVF containing 2 × 10^6^ cells/ml was calculated and added to the complete growth medium. The cells were incubated in a CO_2_ incubator for a total of 14 days or until the cell confluency reached 60-80%, with replacement of the complete growth medium in the flask every 2-3 days. For subculturing the cells, the medium was aspirated and cells were washed once with prewarmed PBS. Cells were detached from the flask by adding 5 ml of TrypLE™ Select CTS™ (Thermo Fisher Scientific, Waltham, MA, USA) and incubated at 37°C for 5 minutes. Upon detachment, 5 ml of PBS was added to the flask and the cell suspension was transferred to a 15 ml conical tube, followed by centrifugation at 200 x g for 5 minutes. The cell pellet was resuspended in a minimal volume of complete growth medium for cell counting. A total of 4 × 10^5^ viable cells were added to a CELLstart™ CTS™ precoated 75 cm^2^ tissue culture flask containing a StemPro® MSC SFM CTS™ complete growth medium, 2% L-glutamine and 1% antibiotics. The cells were incubated in a humidified CO_2_ incubator as above with medium replacement carried out every 2-3 days for optimal cell growth and proliferation.

### 2.6. Multilineage Cell Differentiation

Differentiation of MSC into the adipogenic, chondrogenic, and osteogenic lineages was examined in a representative case. MSCs from passage 2 were harvested using TrypLE™ Select CTS™ and plated in CELLstart™ CTS™ precoated plates in triplicate. Cells plated in 6-well culture plates at 3 × 10^5^ cells/well were used for lineage-specific gene expression studies. Cells plated in 12-well culture plates at 1 × 10^5^ cells/well were used to stain the differentiated cells. The cells were grown in a StemPro® MSC SFM CTS™ complete growth medium until they reached 80% confluency.

#### 2.6.1. Adipogenic Differentiation

The complete growth medium was replaced with DMEM (high glucose, GlutaMAX™ supplement; Thermo Fisher Scientific, Waltham, MA, USA), containing 10% Fetal Bovine Serum, 200 *μ*M indomethacin, 1 *μ*M dexamethasone, 10 *μ*M insulin, and 0.5 mM isobutyl-methyl xanthine (Sigma-Aldrich, St. Louis, MO, USA). This medium was replaced every 2-3 days for optimal differentiation. The plates were incubated in a humidified CO_2_ incubator for 1 week until RNA extraction (6-well plates) and 2 weeks until evaluation for lipid-droplet formation (12-well plates). For visualizing the formation of lipid droplets, cells were fixed in 10% formaldehyde (*v*/*v*) for 10 minutes at room temperature. The fixed cells were washed with 60% isopropanol followed by staining with Oil Red O (Sigma-Aldrich, St. Louis, MO, USA). The stained cells were again washed with 60% isopropanol and counter stained with hematoxylin (Sigma-Aldrich, St. Louis, MO, USA) for staining cell nuclei. The stained cells were washed with distilled water and observed under the light microscope. Images were captured at 40x magnification. Control cells were maintained in a complete growth medium and stained in parallel along with the differentiated cells.

#### 2.6.2. Osteogenic Differentiation

MSCs at 80% confluency were induced to differentiate into osteocytes by replacing the complete growth media with DMEM (high glucose, GlutaMAX™ supplement, Thermo Fisher Scientific, Waltham, MA, USA), containing 10% Fetal Bovine Serum, 50 *μ*M L-ascorbic acid 2-phosphate sesquimagnesium salt hydrate, 0.1 *μ*M dexamethasone, and 10 mM *β*-glycerophosphate (Sigma-Aldrich, St. Louis, MO, USA). The plates were incubated in a humidified CO_2_ incubator with medium replacement every 2-3 days. RNA was extracted from the 6-well plates after 1 week. After 2 weeks of incubation, the cells in the 12-well plates were fixed in 10% formaldehyde (*v*/*v*) for 10 minutes at room temperature. The cells were washed twice with PBS and stained with 2% Alizarin red S solution for 15 minutes at room temperature. The excess stain was removed by washing the cells with distilled water. The stained monolayer was observed under the light microscope, and images were captured at 10x magnification. Control cells were maintained in a complete growth medium and stained in parallel along with the differentiated cells.

#### 2.6.3. Chondrogenic Differentiation

MSCs were induced to differentiate into chondrocytes by replacing the StemPro® MSC SFM CTS™ complete growth medium with the complete StemPro® Chondrogenesis Differentiation medium in 6-well and 12-well culture plates. The medium was replaced every 2-3 days for optimal differentiation. Cells were harvested for RNA extraction after 1 week from the 6-well plates. After 14 days, the cells in the 12-well plates were fixed in 10% formaldehyde (*v*/*v*) for 10 minutes at room temperature. Cells were washed with PBS and stained with 1% Alcian blue solution for 30 minutes at room temperature. The stain was washed off using 3% acetic acid solution followed by rinsing in water. The stained cells were observed under the light microscope, and images were captured at 10x magnification. Control cells were maintained in a complete growth medium and stained in parallel along with the differentiated cells.

### 2.7. Gene Expression: qRT-PCR

Cells were resuspended in TRIzol® Reagent (Sigma-Aldrich, St. Louis, MO, USA), and total RNA was extracted by the phase separation procedure [25]. 1 *μ*g of total RNA was reverse transcribed to cDNA using the qScript™ cDNA SuperMix first-strand synthesis system kit (Quanta Biosciences, Gaithersburg, MD, USA). The cDNA was added to SsoAdvanced™ Universal SYBR® Green Supermix and overlaid onto custom 96-well PCR plates (Bio-Rad Laboratories, Hercules, CA, USA). qRT-PCR was performed using the CFX96™ Real-Time PCR Detection System (Bio-Rad Laboratories, Hercules, CA, USA). Transcripts of the following genes were customized on the 96-well PCR plate to determine the lineage-specific gene expression profile: peroxisome proliferator-activated receptor *γ* (PPAR*γ*), fatty acid desaturase 2 (FADS2), and lipoprotein lipase (LPL) for adipogenic differentiation; integrin-binding sialoprotein (IBSP), runt-related transcription factor (RUNX2), osterix/Sp7 transcription factor (SP7), and beta catenin 1 (CTNNB1) for osteogenic differentiation; and sterol-C4-methyl oxidase-like protein (SC4MOL) and cartilage oligomeric matrix protein (COMP) for chondrogenic differentiation. Glyceraldehyde-3-phosphate dehydrogenase (GAPDH) transcript for each sample was used as an internal control.

### 2.8. Colony-Forming Unit-Fibroblast (CFU-F) Assay

The MesenCult™ Proliferation Kit (Human) (Stemcell Technologies Inc., Vancouver, BC, Canada) was used for the CFU-F assay of adipose tissue MSC. The volume of SVF containing 1 × 10^7^ cells/ml was calculated and added to 15 ml of the prepared MesenCult™ medium containing 1% antibiotic (Penicillin-Streptomycin; Thermo Fisher Scientific, Waltham, MA, USA) and 0.1% blood group “AB” human serum (Corning, Corning, NY, USA) in a 75 cm^2^ tissue culture flask (Falcon®, Corning, Corning, NY, USA). The cells were incubated in a CO_2_ incubator at 37°C for 30 days, with 2 changes in the media during the incubation period, and the growth was evaluated for confluence.

### 2.9. Statistical Analysis

Analysis of the data for both HSC and MSC variables was performed using the means and the standard error of means. Deceased donor data was compared to established living donor ranges. Student's *t*-test (Microsoft Excel) was used to determine the statistical confidence of observed differences. Differences were considered statistically significant at *p* < 0.05.

## 3. Results

### 3.1. Identification of HSC from Deceased Donor Bone Marrow

Iliac crest bone marrow from 12 research-consented deceased donors was procured as per the procedure described in Materials and Methods. The mean collection was 69 ml of liquid bone marrow. Using the BD™SCE single tube assay, we were able to identify the percentage of viable HSC (CD34+ cells) in the iliac crest bone marrow from deceased donors.

**Figure 1:**
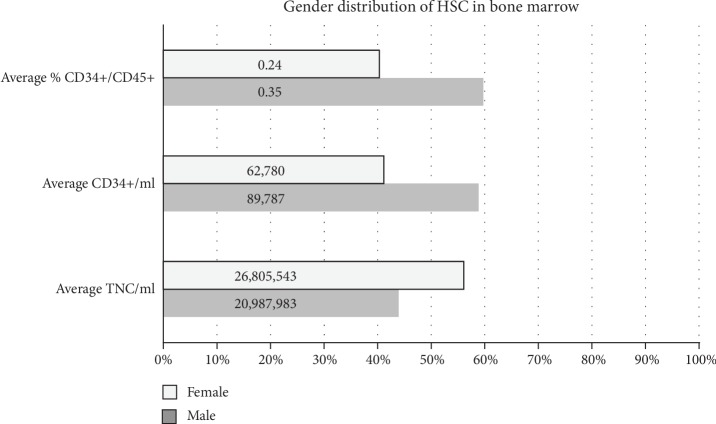
Distribution of hematopoietic stem cells (HSC) (CD34+/CD45+) in deceased donor iliac crest bone marrow based on gender (*n* = 7).

We further investigated the distribution of HSC from iliac crest bone marrow based on the gender and age group in our cohort of deceased donors. As shown in Figure 1, we observed that the TNC/ml was slightly higher in females; however, the percentage of CD34+ cells was lower than in males.

Age of the deceased donor appears to play an important role in the ability to procure viable HSC for regenerative therapy.  Table 2 shows that donors 25 years and younger had the largest number of TNC/ml (CD45+) and the highest percentage of CD34+ cells to CD45+ cells.

### 3.2. Average Percent of HSC

We used published ranges of bone marrow-derived HSC that were isolated from the iliac crest of living donors to compare results.  Table 3 shows the established ranges for living donors and our observed deceased donor means. We observed that the mean values of HSC that we procured from deceased donor iliac crest bone marrow were well within the range of the corresponding values from living donors. This suggests that HSC obtained from deceased donor iliac crest bone marrow are a viable option to living donor HSC.

### 3.3. Colony Formation from Deceased Donor Bone Marrow HSC

**Figure 2:**
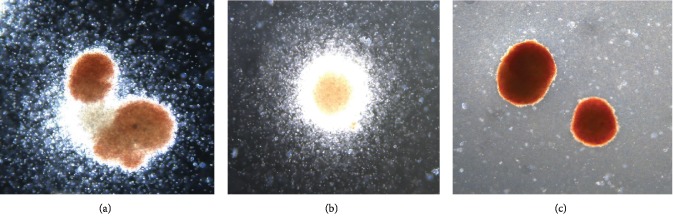
Stem cell colonies grown from the bone marrow from research-consented deceased donors: (a) CFU-GEMM (10x), (b) CFU-GM (10x), and (c) BFU-E (10x).

We performed the CFU assay using the buffy coat isolated from the iliac crest bone marrow of 5 research-consented donors and femur bone marrow of 2 of the research-consented donors to evaluate the colony-forming ability of the HSC. We observed growth of cell colonies after 14 days of incubation (Figures 2). These colonies had a characteristic growth pattern that was identical to the colonies observed from similar samples obtained from living donors.

The number of colonies formed by the HSC from deceased donor iliac crest bone marrow is within the range of established values for HSC from living donor bone marrow. The number of colonies formed by the HSC from the deceased donor femur bone marrow was also observed. As shown in Table 4, we observed that the number of colonies formed by the iliac crest bone marrow was within the range of corresponding values from living donors. We also observed that the number of colonies formed by the femur bone marrow was higher. Furthermore, we observed that the mean number of colonies was higher in male deceased donors and also in deceased donors less than 45 years old. In addition, we observed that the distribution of colonies from the different hematopoietic lineages was similar in bone marrow isolated from the iliac crest and femur.

### 3.4. Timing of Extracting Bone Marrow from Deceased Donors

While performing the extraction of the iliac crest bone marrow from deceased donors, we observed that, although the deceased donors had been heparinized for organ procurement, the timing of extracting the bone marrow was of utmost importance. We found that the bone marrow has to be extracted within 2 hours after pronouncement of death since the bone marrow begins to coagulate and solidify within the iliac crest bone. It may be for this reason that the volume of bone marrow obtained from deceased donors was less than that obtained from living donors. In living donor extraction, there is continuous circulation of blood through the bone marrow and the bone marrow remains in a liquid form.

The bone marrow located within the femur is in a solid state and can be scooped out or flushed out using heparin. We did not notice any time constraints for femur bone marrow procurement.

### 3.5. Adipose Tissue-Derived MSC from Deceased Donors

**Figure 3:**
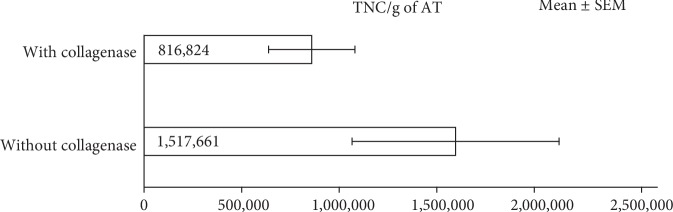
Comparison of mean total nucleated cells in the SVF per gram (TNC/g) of adipose tissue when treated with and without collagenase (*n* = 6, *p* value = 0.025).

We procured adipose tissue from 27 research-consented donors obtaining between 45 and 876 grams of adipose tissue from each donor. This volume can be significantly higher based on the body mass index of the donor. Of the 27 donors, 11 donor samples were processed using enzymatic digestion (collagenase), 9 donor samples were processed using the AC:Px® System (without collagenase treatment), 6 donor samples were processed using both methods, and 1 donor sample was not processed.  Figure 3 shows the mean values of TNC in the SVF/g of adipose tissue from samples processed both with and without enzymatic digestion. We observed that we could procure a significantly larger number of TNC in the SVF/g of adipose tissue from samples processed using the AC:Px® System with no enzyme treatment as compared to enzymatic digestion.

**Figure 4:**
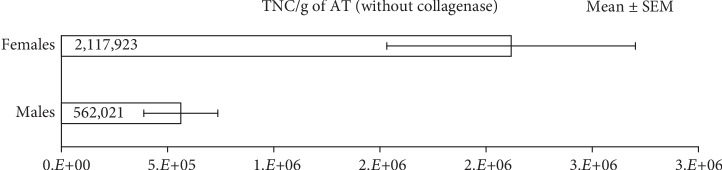
Comparison of mean total nucleated cells in the SVF per gram (TNC/g) of adipose tissue when treated without collagenase between females (*n* = 5, mean age 58, mean BMI 30) and males (*n* = 8, mean age 40, mean BMI 32) (*p* value = 0.00006).

Interestingly, we also observed that adipose tissue from female deceased donors yielded a much greater number of TNC in the SVF/g of adipose tissue as compared to males as shown in Figure 4. Both the male and female groups had a similar mean body mass index (BMI). The average age for the female cohort was higher than the average age for the male cohort in our study.

**Figure 5:**
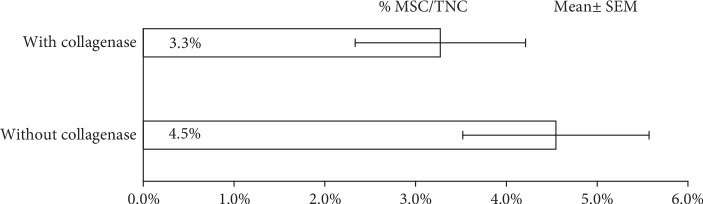
Percentage of mesenchymal stem cells per total nucleated cells (MSC/TNC) in the SVF of adipose tissue when treated with or without collagenase (*n* = 11).

We analyzed the presence of MSC in the SVF using the established cell surface markers for MSC. The fraction of cells exhibiting a CD29, CD44, CD73, CD90, and CD105 phenotype accompanied with a lack of expression of CD45 and CD11b was assessed using flow cytometry. We observed that the cell surface marker CD105 was minimally expressed in the native, primary cell suspension of deceased donor MSC [20, 26]. The percentage of MSC/TNC was higher in adipose tissue that was processed without enzymatic digestion (Figure 5); however, it was not statistically different.

The mean TNC in the SVF/g of adipose tissue obtained from the deceased donor samples was compared to established values observed in living donors. We observed that the mean TNC in the SVF/g of adipose tissue of samples processed with and without collagenase fell above the living donor value ranges (Table 5). The mean %MSC/TNC value fell within the living donor value range.

**Figure 6:**
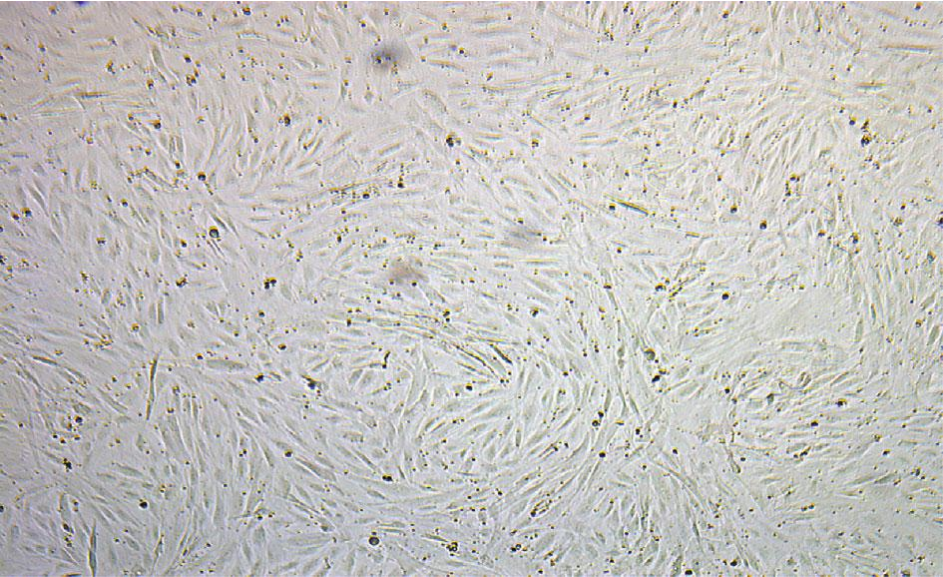
Growth to confluency of deceased donor adipose-derived MSC (CFU-F).

We performed the CFU assay as described in Materials and Methods. We used 4 samples of SVF isolated from the research-consented deceased donor adipose tissues that were processed without enzymatic digestion to evaluate the colony-forming ability of the MSC. We observed growth from all 4 samples. A representative image of the growth confluence of the deceased donor adipose-derived MSC is shown in Figure 6.

### 3.6. In Vitro Differentiation of MSC from Deceased Donors

**Figure 7:**
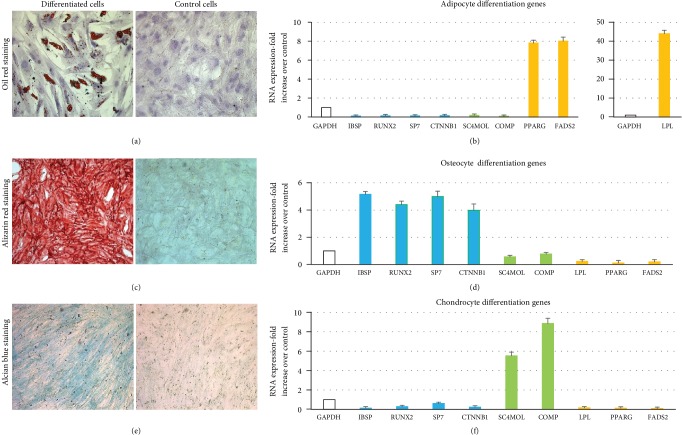
Differentiation of MSC isolated from non-enzyme-treated SVF. Light microscopic images are representative of three separate experiments. (a) Adipocyte differentiation. (c) Osteocyte differentiation. (e) Chondrocyte differentiation. (b, d, f) Semiquantitative qRT-PCR analysis showing lineage-specific gene expression.

The functionality of the MSC isolated from one of the non-enzyme-treated SVF fraction was analyzed by examining their multilineage differentiation potential using standard in vitro tissue culture techniques. MSCs were induced to differentiate into adipocytes, osteocytes, and chondrocytes by replacing the growth medium with a lineage-specific medium. We observed that induction of adipocyte differentiation led to the morphological conversion of MSC to form lipid droplets, a characteristic of mature white adipocytes (Figure 7(a)). This was further confirmed by analyzing the increased expression of specific genes related to adipocyte differentiation such as peroxisome proliferator-activated receptor *γ* (PPAR*γ*), fatty acid desaturase 2 (FADS2), and lipoprotein lipase (LPL) (Figure 7(b)). Similarly, induction under osteogenic conditions led to the successful differentiation of MSC into osteocytes (Figure 7(c)). We also observed increased expression of specific osteogenic lineage genes such as integrin-binding sialoprotein (IBSP), runt-related transcription factor (RUNX2), osterix/Sp7 transcription factor (SP7), and beta catenin 1 (CTNNB1) (Figure 7(d)). In addition, MSC differentiated into chondrocytes when exposed to a specific chondrocyte differentiation medium (Figure 7(e)). This was accompanied with the increased expression of specific genes associated with chondrocyte differentiation such as sterol-C4-mehtyl oxidase-like protein (SC4MOL) and cartilage oligomeric matrix protein (COMP) (Figure 7(f)). In all cases, the undifferentiated cells did not exhibit any changes at either the morphological or the gene expression level.

## 4. Discussion

Living donor HSC is currently used to treat patients with disorders affecting the hematopoietic system that are inherited, acquired, or result from myeloablative treatment. According to the United States Institute for Justice, at any time, approximately 7,500 Americans are searching for an unrelated HSC donor [27]. This is made more challenging due to a large racial/ethnic disparity in available HSC donors, making it difficult for minority and mixed-race patients awaiting a HSC transplant to find a suitable match.

In addition, MSCs are being evaluated in clinical studies for their use to repair, replace, restore, or regenerate cells in the body. Both HSC and MSC are restricted by the number of available living donors and quantity of tissue procured.

The acceptance of deceased donor stem cells as a putative cell source for therapeutic applications would be advantageous to patients awaiting stem cell therapies. The routine process of organ/tissue procurement from deceased donors could be expanded to include collection of HSC and MSC. Deceased donors are routinely phenotyped for human leukocyte antigens and evaluated for the presence of any potential infectious diseases, thus making them a safe, accessible, and an economically viable source of stem cells. These stem cells could be expanded and/or cryopreserved and banked for future application [28].

Our initial efforts in procuring and characterizing the HSC from deceased donor bone marrow have been very promising. It is well known that the quantity of the CD34+ cells is an important dosage indicator for the clinical success of HSC cell therapy [29]. In particular, cell count parameters such as TNC/ml and %CD34+/CD45+ cells in the bone marrow are acceptable indicators for the suitability of the procured bone marrow from living donors for HSC therapy. After analyzing these same parameters in the bone marrow procured from research-consented deceased donors, we observed that the mean values for these same parameters were well within the published living donor ranges. Our data suggest that HSC from deceased donor bone marrow may be suited for the same application as living donor HSC since they have the same clinically acceptable quantities for cell-based therapy. In addition, we observed that the colony-forming ability of deceased donor iliac crest-derived bone marrow HSC was similar to living donors. Interestingly, the deceased donor femur-derived bone marrow HSC grew a greater number of colonies than the iliac crest-derived bone marrow. In addition, our observations suggest that younger deceased donor bone marrow and bone marrow from males yield a higher number of colonies than older deceased donor bone marrow and bone marrow from females. Our studies suggest that the deceased donor HSC are functionally viable and suitable for regenerative therapy applications. Additionally, femur-derived bone marrow may be an alternative to iliac crest-derived bone marrow. Furthermore, studies evaluating vertebrae from cadaveric donors suggest additional sources for obtaining bone marrow [30]. These additional solid bone marrow sources may have the advantage of obtaining viable stem cells after an extended period of storage.

There is a caveat to procuring deceased donor liquid bone marrow. We observed that there was a limitation on the timing of extraction of the iliac crest bone marrow from deceased donors. Since there is no circulation of blood through the bone marrow in deceased donors, the bone marrow begins to coagulate within 2 hours of death. This leaves only a small window of opportunity to procure a significant volume of bone marrow after pronouncement of death. In addition, the volume of iliac crest bone marrow obtained from deceased donors is also smaller than living donors.

The yield and percentage of HSC from bone marrow are also dependent on the donor characteristics [31, 32]. The influence of variables such as gender and age on the clinical parameters of HSC has been observed [33, 34]. It has been reported that the number of CD34+ cells from bone marrow derived from vertebral bodies is lower in females [33]. Further, the median CD34+ cell concentration in male infants was higher than female infants [35]. It has also been suggested that sex hormones may have an effect on HSC and hematopoiesis [35]. Our observations that the number of CD34+ cells in iliac crest bone marrow from deceased donor females was less than that of males are in agreement with published results. The lower CD34+ HSC in females might be due to the influence of female sex hormones that have a pronounced effect on hematopoiesis in the bone marrow of females as suggested by Ray et al. [36].

We have assessed the influence of age on the quantity and viability of CD34+ HSC in deceased donors. We observed that donors 25 years and younger had a higher percentage of CD34+ cells, suggesting that bone marrow from younger deceased donors would be a preferred source of functionally relevant HSC. In addition, Stolzing et al. suggest that there is a notable decrease in bone marrow-derived MSCs with age [37]. There are conflicting reports in the literature on the influence of age on CD34+ cell count. Some of the reports suggest that there is indeed a decrease in the CD34+ cell count with increasing age [38, 39], while others suggest that although there is not much difference in the HSC number with increasing age, the functionality decreases as age increases [40, 41]. Overall, our observation related to the influence of age on the number and functionality of HSC procured from deceased donors is in accordance with the published literature.

Adipose tissue provides a rich source of MSC which can be easily isolated from the SVF. These MSCs are a readily available source for use in tissue engineering and regenerative medicine therapies. Adipose tissue is present in large quantities and is obtained from living donors using the invasive procedure of liposuction [42]. In addition to the pain and associated discomfort to the living donor, the liposuction procedure can potentially damage the SVF cells, resulting in lower frequencies of viable cells [43]. Standard processing of lipoaspirate from living donors utilizes enzymatic digestion to isolate the SVF. Published yields of the total viable nucleated cell count in the SVF using the enzymatic isolation technique range from 1.0 × 10^5^ to 1.3 × 10^6^ cells/cc of lipoaspirate [44, 45]. Of these, only 5% of cells were found to be MSC [46]. Mechanical nonenzymatic methods for SVF isolation from living donor lipoaspirates have shown significant lower yields of TNC, ranging from 1.0 × 10^4^ to 2.4 × 10^5^ cells/cc of lipoaspirate [47] with 5% of these being MSC [48]. The lower yield of cells from the mechanical nonenzymatic isolation techniques has been attributed to the location of MSC in the perivascular space of adipose tissue. Mechanical shearing of adipose tissue does not disrupt the extracellular matrix as compared to the enzymatic method, leaving the MSC trapped within the vascular endothelial layer and connective tissue fragments in the lipoaspirate [49, 50].

Recently published literature demonstrates isolation of MSC from abdomen-derived, solid adipose tissue [51–53]. Studies have also shown that cadaveric adipose tissue can be used as a source of MSC [54]. We have used solid adipose tissue that was excised from the abdomen of research-consented deceased donors. We compared two techniques to isolate the SVF from this tissue. The first technique used a combination of mechanical mincing (using the AC:Px® System) and enzymatic digestion method. The second technique utilized only mechanical mincing of adipose tissue using the AC:Px® System without enzymatic digestion. We observed that the nonenzymatic method had a greater number of total viable nucleated cells per gram of solid adipose tissue (15.2 × 10^5^ cells/g) and a higher yield of MSC (4.5%) as compared to the enzymatic digestion method (8.2 × 10^5^ cells/g) with only 3.3% of these cells as MSC. Our observations suggest that nonenzymatic mechanical mincing of solid adipose tissue using the AC:Px® System released a greater number of TNC and a higher percentage of MSC as compared to earlier studies. Further, enzymatic treatment of the minced tissue reduced the number of TNC and MSC. Similar results have been observed with other solid tissues, including umbilical cord tissue. One possible rationale for this observation is that enzymatic digestion of the adipose tissue possibly induces cell death leading to fewer cells being isolated [55]. Numerous investigators have detected MSC in the SVF of adipose tissue by using a series of antibodies that bind to MSC surface epitopes [7, 23]. There is considerable heterogeneity in the different cell surface markers reported for MSC. Some of this heterogeneity might be due to the presence of a mixed population of cells or the modulation of cell surface proteins during cell culture. Identification of MSC immediately after isolation of SVF from adipose tissue would help discriminate between markers that are expressed *in vivo* from those that are expressed only after in vitro manipulation. We have used antibodies against the most commonly expressed epitopes on MSC to identify them in the SVF immediately after isolation from the adipose tissue. Our observations suggest that nonenzymatic isolation of SVF from adipose tissue would be the most advantageous method to get the highest yield of MSC. In addition, we also observed that adipose tissue from deceased donor females was a better source for MSC as it resulted in a higher yield of TNC in the SVF/g of adipose tissue compared to deceased donor males.

An important aspect of MSC for their clinical application in regenerative medicine is their multipotent differentiation capacity [56]. MSCs derived from adipose tissue have the capacity to differentiate into adipocytes, chondrocytes [57], and osteoblasts [58]. We confirmed the versatile trilineage differentiation potential of deceased donor-derived MSC isolated from the nonenzymatic SVF fraction by exposing them to differentiation media specific for each cell type. Adipocyte differentiation led to the production of abundant lipid vacuoles along with the elevated expression of adipocyte-associated genes (LPL, PPAR*γ*, and FADS2). Staining of cells with Alizarin red, an early stage marker of matrix mineralization and indicator of calcific deposition, was observed when MSCs were induced to differentiate into osteocytes. Genes representing early phase of osteogenesis differentiation (IBSP, RUNX2, SP7, and CTNNB1) showed an increase in expression over undifferentiated cells. Similarly, we observed chondrogenic differentiation of MSC indicated by Alcian blue staining of cartilage matrix in the differentiated cells and associated increase in the expression of SC4MOL and COMP genes that are indicators of chondrogenesis.

## 5. Conclusion

We have established that deceased donor stem cells are similar in number and function to living donor stem cells. Our results show that deceased donor stem cells have the same functional ability to form colonies and retain their multilineage differentiation potential as living donor stem cells. The deceased donor stem cells can be routinely procured and potentially supplement the current available living donor stem cell sources.

We have recently equipped our laboratory to process and produce cells in agreement with good manufacturing practice-compliant (GMP) standards in preparation for future clinical scale expansion and banking. We plan to evaluate the bone marrow and adipose tissue procured from a larger number of deceased donors that range in age, race, and cause of death. In addition, we plan to examine other deceased donor tissue sources for MSC presence and differentiation potential. Evaluation of a wider pool of deceased donors will help us understand the variations in the number and functionality of HSC and MSC. This information will help us identify a population of potential deceased donors from whom we could obtain the maximum number of viable, functional HSC and MSC.

## Figures and Tables

**Table 1:** Patient demographics.

Patient #	Age	Gender	Race	Cause of death	Iliac crest bone marrow	Adipose tissue	Femur bone marrow
1	41	F	Hispanic	Stroke	X	X	
2	39	M	Black	Homicide	X		
3	25	M	Caucasian	Stroke	X	X	
4	13	F	Black	Motor vehicle accident	X		
5	26	M	Caucasian	Stroke	X		X
6	64	M	Hispanic	Stroke	X		
7	35	F	Caucasian	Natural causes	X	X	
8	21	M	Caucasian	Suicide	X		
9	43	M	Hispanic	Stroke		X	
10	35	F	Black	Motor vehicle accident		X	
11	40	F	Hispanic	Motor vehicle accident		X	
12	19	F	Hispanic	Suicide		X	
13	62	M	Caucasian	Stroke		X	
14	51	F	Black	Cardiac arrest		X	
15	38	M	Hispanic	Head trauma		X	
16	24	M	Caucasian	Suicide		X	
17	52	F	Caucasian	Head trauma		X	
18	69	F	Caucasian	Stroke		X	
19	32	M	South Asian	Natural causes		X	
20	43	M	Caucasian	Cardiac arrest		X	
21	52	F	Hispanic	Natural causes		X	
22	39	M	Hispanic	Head trauma		X	
23	42	M	Caucasian	Suicide		X	
24	49	F	Black	Stroke	X	X	
25	51	M	Hispanic	Head trauma	X	X	X
26	31	M	Caucasian	Drug intoxication	X	X	
27	46	M	Black	Cardiac arrest		X	
28	25	M	Hispanic	Homicide		X	
29	64	F	Caucasian	Stroke		X	
30	50	M	Hispanic	Natural causes		X	
31	26	M	Caucasian	Natural causes	X		
32	44	F	Hispanic	Natural causes		X	
33	62	F	Hispanic	Stroke		X	

**Table 2:** Distribution of HSC in deceased donor iliac crest bone marrow based on age (*n* = 7).

Age group	Ave TNC (ml)	Ave CD34+ (ml)	Ave %CD34+ of CD45+
Age ≤ 25 years (*n* = 2)	3.08 × 10^7^	1.75 × 10^5^	0.50
Age 26-45 years (*n* = 4)	1.92 × 10^7^	0.50 × 10^5^	0.27
Age > 45 years (*n* = 1)	2.03 × 10^7^	0.27 × 10^5^	0.13

**Table 3:** Comparison of the HSC procured from living and deceased donor iliac crest bone marrow.

Bone marrow: iliac crest (*n* = 7)	Living donor	Deceased donor (mean)	Standard error of mean
TNC (ml)	11 × 10^6^–34 × 10^6^ [59]	23 × 10^6^	3.3 × 10^6^
CD34+ (ml)	0.05 × 10^6^–0.46 × 10^6^ [59]	0.09 × 10^6^	0.03 × 10^6^

**Table 4:** Colony-forming units of HSC procured from living and deceased donor bone marrow.

	Living donor	Deceased donor (mean)	Standard error of mean
*Bone marrow: iliac crest (n* = 5)			
CFU (per 2 × 10^5^)	49–722 [60]	400	119
<45 years (*n* = 3)		512	
>45 years (*n* = 2)		232	
Male (*n* = 3)		443	
Female (*n* = 2)		336	
*Hematopoietic lineages: 26% CFU-GM, 6% CFU-GEMM, 68% BFU-E*			
*Bone marrow: femur (n* = 2)			
CFU (per 2 × 10^5^)	N/A	1,152	136
*Hematopoietic lineages: 29% CFU-GM, 5% CFU-GEMM, 66% BFU-E*			

**Table 5:** MSC from adipose tissue: comparison of MSC isolated from living and deceased donors.

Adipose	Living donor SVF	Deceased donor SVF			
		With collagenase		Without collagenase	
		Mean	Standard error of mean	Mean	Standard error of mean
TNC/g	1.8 × 10^5^–5.4 × 10^5^ [61]	8.2 × 10^5^	2.4 × 10^5^	15.2 × 10^5^	5.8 × 10^5^
%MSC/TNC	1-10% [62]	3.3%	1%	4.5%	1%

## Data Availability

The data used to support the findings of this study are included within the article.

## References

[1] Harris D. T. (2014). Stem cell banking for regenerative and personalized medicine. *Biomedicine*.

[2] Gimble J. M. (2003). Adipose tissue-derived therapeutics. *Expert Opinion on Biological Therapy*.

[3] Stocchero I. N., Stocchero G. F., Sterodimas Y.-G. I. A. (2011). Isolation of stem cells from human adipose tissue: technique, problems, and pearls. *Adipose Stem Cells and Regenerative Medicine*.

[4] National Marrow Donor Program HLA matching. https://bethematch.org/for-patients-and-families/finding-a-donor/hla-matching/.

[5] National Marrow Donor Program (2015). Transplant basics. https://bethematch.org/transplant-basics/.

[6] Lagasse E., Shizuru J. A., Uchida N., Tsukamoto A., Weissman I. L. (2001). Toward regenerative medicine. *Immunity*.

[7] Pittenger M. F., Mackay A. M., Beck S. C. (1999). Multilineage potential of adult human mesenchymal stem cells. *Science*.

[8] Fraser J. K., Wulur I., Alfonso Z., Hedrick M. H. (2006). Fat tissue: an underappreciated source of stem cells for biotechnology. *Trends in Biotechnology*.

[9] Harasymiak-Krzyżanowska I., Niedojadło A., Karwat J. (2013). Adipose tissue-derived stem cells show considerable promise for regenerative medicine applications. *Cellular & Molecular Biology Letters*.

[10] Orbay H., Tobita M., Mizuno H. (2012). Mesenchymal stem cells isolated from adipose and other tissues: basic biological properties and clinical applications. *Stem Cells International*.

[11] Gimble J., Guilak F. (2003). Adipose-derived adult stem cells: isolation, characterization, and differentiation potential. *Cytotherapy*.

[12] Ray R. N., Cassell M., Chaplin H. (1961). A new method for the preparation of human cadaver bone marrow for transfusion. *Blood*.

[13] Rao A. S., Fontes P., Zeevi A. (1994). Combined bone marrow and whole organ transplantation from the same donor. *Transplantation Proceedings*.

[14] Mansilla E., Mártire K., Roque G. (2013). Salvage of cadaver stem cells (CSCs) as a routine procedure: history or future for regenerative medicine. *Journal of Transplantation Technologies & Research*.

[15] Laywell E. D., Kukekov V. G., Steindler D. A. (1999). Multipotent neurospheres can be derived from forebrain subependymal zone and spinal cord of adult mice after protracted postmortem intervals. *Experimental Neurology*.

[16] Xu Y., Kimura K., Matsumoto N., Ide C. (2003). Isolation of neural stem cells from the forebrain of deceased early postnatal and adult rats with protracted post-mortem intervals. *Journal of Neuroscience Research*.

[17] Michalova J., Savvulidi F., Sefc L., Forgacova K., Necas E. (2011). Cadaveric bone marrow as potential source of hematopoietic stem cells for transplantation. *Chimerism*.

[18] Bender J. G., Unverzagt K., Walker D. E. (1994). Phenotypic analysis and characterization of CD34+ cells from normal human bone marrow, cord blood, peripheral blood, and mobilized peripheral blood from patients undergoing autologous stem cell transplantation. *Clinical Immunology and Immunopathology*.

[19] Dominici M., Le Blanc K., Mueller I. (2006). Minimal criteria for defining multipotent mesenchymal stromal cells. The International Society for Cellular Therapy position statement. *Cytotherapy*.

[20] Locke M., Windsor J., Dunbar P. R. (2009). Human adipose-derived stem cells: isolation, characterization and applications in surgery. *ANZ Journal of Surgery*.

[21] Zimmerlin L., Donnenberg V. S., Rubin J. P., Donnenberg A. D. (2013). Mesenchymal markers on human adipose stem/progenitor cells. *Cytometry. Part A*.

[22] Boquest A. C., Shahdadfar A., Frønsdal K. (2005). Isolation and transcription profiling of purified uncultured human stromal stem cells: alteration of gene expression after in vitro cell culture. *Molecular Biology of the Cell*.

[23] Gronthos S., Franklin D. M., Leddy H. A., Robey P. G., Storms R. W., Gimble J. M. (2001). Surface protein characterization of human adipose tissue-derived stromal cells. *Journal of Cellular Physiology*.

[24] Puissant B., Barreau C., Bourin P. (2005). Immunomodulatory effect of human adipose tissue-derived adult stem cells: comparison with bone marrow mesenchymal stem cells. *British Journal of Haematology*.

[25] Chomcynski P., Mackay K. (1995). Short technical reports. Modification of the TRI reagent procedure for isolation of RNA from polysaccharide- and proteoglycan-rich sources. *Biotechniques*.

[26] Taghizadeh R. R., Cetrulo K. J., Cetrulo C. L. (2011). Wharton’s jelly stem cells: future clinical applications. *Placenta*.

[27] Bone marrow statistics. http://ij.org/bonemarrowstatistics/.

[28] Lechanteur C., Briquet A., Giet O., Delloye O., Baudoux E., Beguin Y. (2016). Clinical-scale expansion of mesenchymal stromal cells: a large banking experience. *Journal of Translational Medicine*.

[29] Yasuhara S., Yasunaga Y., Hisatome T. (2010). Efficacy of bone marrow mononuclear cells to promote bone regeneration compared with isolated CD34+ cells from the same volume of aspirate. *Artificial Organs*.

[30] Gorantla V. S., Schneeberger S., Moore L. R. (2012). Development and validation of a procedure to isolate viable bone marrow cells from the vertebrae of cadaveric organ donors for composite organ grafting. *Cytotherapy*.

[31] Bouwmeester W., Fechter M. M., Heymans M. W., Twisk J. W., Ebeling L. J., Brand A. (2010). Prediction of nucleated cells in bone marrow stem cell products by donor characteristics: a retrospective single centre analysis. *Vox Sanguinis*.

[32] Zhang C., Chen X.-H., Zhang X. (2010). Stem cell collection in unmanipulated HLA-haploidentical/mismatched related transplantation with combined granulocyte-colony stimulating factor-mobilised blood and bone marrow for patients with haematologic malignancies: the impact of donor characteristics and procedural settings. *Transfusion Medicine*.

[33] Newman H., Reems J. A., Rigley T. H., Bravo D., Strong D. M. (2003). Donor age and gender are the strongest predictors of marrow recovery from cadaveric vertebral bodies. *Cell Transplantation*.

[34] de Haan G., Van Zant G. (1999). Dynamic changes in mouse hematopoietic stem cell numbers during aging. *Blood*.

[35] Aroviita P., Teramo K., Hiilesmaa V., Kekomaki R. (2005). Cord blood hematopoietic progenitor cell concentration and infant sex. *Transfusion*.

[36] Ray R., Novotny N. M., Crisostomo P. R., Lahm T., Abarbanell A., Meldrum D. R. (2008). Sex steroids and stem cell function. *Molecular Medicine*.

[37] Stolzing A., Jones E., McGonagle D., Scutt A. (2008). Age-related changes in human bone marrow-derived mesenchymal stem cells: consequences for cell therapies. *Mechanisms of Ageing and Development*.

[38] Vaziri H., Dragowska W., Allsopp R. C., Thomas T. E., Harley C. B., Lansdorp P. M. (1994). Evidence for a mitotic clock in human hematopoietic stem cells: loss of telomeric DNA with age. *Proceedings of the National Academy of Sciences of the United States of America*.

[39] Stelzer I., Fuchs R., Schraml E. (2010). Decline of bone marrow-derived hematopoietic progenitor cell quality during aging in the rat. *Experimental Aging Research*.

[40] Berkahn L., Keating A. (2004). Hematopoiesis in the elderly. *Hematology*.

[41] Morrison S. J., Wandycz A. M., Akashi K., Globerson A., Weissman I. L. (1996). The aging of hematopoietic stem cells. *Nature Medicine*.

[42] Dolsky R. L. (1984). Body sculpturing by lipo-suction extraction. *Aesthetic Plastic Surgery*.

[43] Aust L., Devlin B., Foster S. J. (2004). Yield of human adipose-derived adult stem cells from liposuction aspirates. *Cytotherapy*.

[44] Suga H., Eto H., Aoi N. (2010). Adipose tissue remodeling under ischemia: death of adipocytes and activation of stem/progenitor cells. *Plastic and Reconstructive Surgery*.

[45] Yoshimura K., Shigeura T., Matsumoto D. (2006). Characterization of freshly isolated and cultured cells derived from the fatty and fluid portions of liposuction aspirates. *Journal of Cellular Physiology*.

[46] Raposio E., Caruana G., Bonomini S., Libondi G. (2014). A novel and effective strategy for the isolation of adipose-derived stem cells: minimally manipulated adipose-derived stem cells for more rapid and safe stem cell therapy. *Plastic and Reconstructive Surgery*.

[47] Markarian C. F., Frey G. Z., Silveira M. D. (2014). Isolation of adipose-derived stem cells: a comparison among different methods. *Biotechnology Letters*.

[48] Baptista L. S., do Amaral R. J. F. C., Carias R. B. V., Aniceto M., Claudio-da-Silva C., Borojevic R. (2009). An alternative method for the isolation of mesenchymal stromal cells derived from lipoaspirate samples. *Cytotherapy*.

[49] Zimmerlin L., Donnenberg V. S., Pfeifer M. E. (2010). Stromal vascular progenitors in adult human adipose tissue. *Cytometry. Part A*.

[50] Baer P. C., Geiger H. (2012). Adipose-derived mesenchymal stromal/stem cells: tissue localization, characterization, and heterogeneity. *Stem Cells International*.

[51] Ardeshirylajimi A., Rafeie F., Zandi-Karimi A. (2016). Fat harvesting site is an important determinant of proliferation and pluripotency of adipose-derived stem cells. *Biologicals*.

[52] Schneider S., Unger M., van Griensven M., Balmayor E. R. (2017). Adipose-derived mesenchymal stem cells from liposuction and resected fat are feasible sources for regenerative medicine. *European Journal of Medical Research*.

[53] Jurgens W. J. F. M., Oedayrajsingh-Varma M. J., Helder M. N. (2008). Effect of tissue-harvesting site on yield of stem cells derived from adipose tissue: implications for cell-based therapies. *Cell and Tissue Research*.

[54] Shi Y., Niedzinski J. R., Samaniego A., Bogdansky S., Atkinson B. L. (2012). Adipose-derived stem cells combined with a demineralized cancellous bone substrate for bone regeneration. *Tissue Engineering. Part A*.

[55] Taghizadeh R. R., Cetrulo K. J., Cetrulo C. L. (2018). Collagenase impacts the quantity and quality of native mesenchymal stem/stromal cells derived during processing of umbilical cord tissue. *Cell Transplantation*.

[56] Rohban R., Pieber T. R. (2017). Mesenchymal stem and progenitor cells in regeneration: tissue specificity and regenerative potential. *Stem Cells International*.

[57] Zuk P. A., Zhu M., Mizuno H. (2001). Multilineage cells from human adipose tissue: implications for cell-based therapies. *Tissue Engineering*.

[58] Halvorsen Y. C., Wilkison W. O., Gimble J. M. (2000). Adipose-derived stromal cells—their utility and potential in bone formation. *International Journal of Obesity*.

[59] Lannert H., Able T., Becker S. (2008). Optimizing BM harvesting from normal adult donors. *Bone Marrow Transplantation*.

[60] Gee A. P. (1991). Colony assays. *Bone Marrow Processing and Purging*.

[61] Fraser J. K., Hicok K. C., Shanahan R., Zhu M., Miller S., Arm D. M. (2014). The Celution® system: automated processing of adipose-derived regenerative cells in a functionally closed system. *Advances in Wound Care*.

[62] Bourin P., Bunnell B. A., Casteilla L. (2013). Stromal cells from the adipose tissue-derived stromal vascular fraction and culture expanded adipose tissue-derived stromal/stem cells: a joint statement of the International Federation for Adipose Therapeutics and Science (IFATS) and the International Society for Cellular Therapy (ISCT). *Cytotherapy*.

